# Clinical importance of [^18^F]fluorodeoxyglucose positron emission tomography/computed tomography in the management of patients with bronchoalveolar carcinoma: Role in the detection of recurrence

**DOI:** 10.3892/ol.2013.1257

**Published:** 2013-03-14

**Authors:** EVANGELIA SKOURA, IOANNIS E. DATSERIS, DIMITRIOS EXARHOS, SOPHIA CHATZIIOANNOU, GEORGIOS OIKONOMOPOULOS, ALEXANDROS SAMARTZIS, CHARIKLIA GIANNOPOULOU, KONSTANTINOS N. SYRIGOS

**Affiliations:** 1 Departments of Nuclear Medicine, Evangelismos General Hospital, Athens, Greece; 2 Computed Tomography, Evangelismos General Hospital, Athens, Greece; 3 Department of Nuclear Medicine, Biomedical Research Foundation, Academy of Athens, Athens, Greece; 4 Oncology Unit, Third Department of Medicine, Athens Medical School, Sotiria General Hospital, Athens, Greece

**Keywords:** bronchoalveolar carcinoma, non-small cell lung cancer, positron emission tomography, [^18^F]fluorodeoxyglucose, standardized uptake value

## Abstract

[^18^F]fluorodeoxyglucose (FDG) positron emission tomography (PET)/computed tomography (CT) has been reported to have a low sensitivity in the initial diagnosis of bronchoalveolar carcinoma (BAC) due to BAC’s low metabolic activity. The aim of this study was to assess the value of [^18^F]FDG-PET/CT in the detection of BAC recurrence. Between February 2007 and September 2011, the [^18^F]FDG-PET/CT scans that were performed on patients with known, histologically proven BAC were studied. A total of 24 [^18^F]FDG-PET/CT scans were performed in 22 patients, including 16 males and 6 females, with a mean age of 65±9 years. Among the scans, 15 were performed to assess for possible recurrence with equivocal findings in conventional imaging methods and 9 for restaging post-therapy. In all cases conventional imaging studies (CT and MRI) were performed 5–30 days prior to PET/CT. Among the 24 [^18^F]FDG-PET/CT scans, 18 were positive and 6 negative. Among the 15 [^18^F]FDG-PET/CT scans performed for suspected recurrence, 34 lesions were detected and the mean maximum standardized uptake value (SUVmax) was 6.8±3.26. In nine scans, upstaging was observed, while two were in agreement with the findings of the conventional modalities. A greater number of lesions were detected in two scans and fewer lesions were detected in one, with no change in staging. Only one scan was negative. By contrast, in patients examined for restaging, there were only five lesions with a mean SUVmax of 4.86±3.18. Agreement between the findings of [^18^F]FDG-PET/CT and the conventional modalities was observed in 8 out of 9 cases. Although [^18^F]FDG-PET/CT has been reported to have a low sensitivity in the initial diagnosis of BAC, the present results indicate that when there is recurrence, the lesions become [^18^F]FDG avid. [^18^F]FDG-PET/CT may provide further information in patients evaluated for recurrence and thus improve patient management.

## Introduction

The term bronchioloalveolar lung carcinoma (BAC) was first applied in 1960 to describe peripheral, well-differentiated lung tumors that grew in a lepidic manner without distortion of the lung architecture (1–3). In 1999, the World Health Organization (WHO) established the more restrictive definition of BAC as tumors with a pure lepidic spreading pattern and no evidence of stromal, vascular or pleural invasion (4,5). This definition was retained in the 2004 classification. According to this definition, BAC is a carcinoma *in situ* and a tumor cannot be classified as BAC if it is associated with lymphatic or systemic metastases (4,6).

Positron-emission tomography (PET) utilizing the radio-labelled glucose analog fluorine-18 (^18^F) fluorodeoxyglucose, (FDG-PET) is a widely accepted imaging method in oncology. The radiopharmaceutical [^18^F]FDG is transported as a glucose analog and subsequently phosphorylated and trapped within cancer cells, thus enabling the evaluation of glucose metabolism with PET (7). It is known that the majority of malignant tumors have a higher rate of glucose metabolism than normal tissue and therefore exhibit higher FDG uptake than background tissue. Numerous studies have demonstrated that the uptake of [^18^F]FDG is associated with tumor grade and proliferation status in a wide variety of tumors (8). Consequently, [^18^F]FDG uptake varies widely, depending on the histological type and aggressiveness of the tumor (8).

[^18^F]FDG-PET/CT has become an established method for staging patients with non-small cell lung cancer (NSCLC). It has a sensitivity of ∼98% in most series of lung cancer cases, but in the literature [^18^F]FDG-PET/CT has been reported to have a low sensitivity in the initial diagnosis of BAC due to BAC’s low metabolic activity (9). This is why BAC was considered to be one of the main reasons for false negative findings when [^18^F]FDG-PET/CT is used to assess solitary pulmonary nodules (10,11).

At present, there are no published data in the worldwide literature concerning the clinical usefulness of [^18^F]FDG-PET/CT in the management of patients with confirmed BAC rather than at the stage of the initial diagnosis. The purpose of the present study is to assess the value of [^18^F]FDG-PET/CT in the detection of BAC recurrence and restaging following treatment and to evaluate [^18^F]FDG-PET/CT uptake at the sites of metastasis and recurrence.

## Materials and methods

### Patients

This is a retrospective analysis of all the [^18^F]FDG-PET/CT studies performed on patients with known, histologically confirmed BAC between February 2007 and September 2011. This study was approved by the Ethics Committee of the Evangelismos General Hospital, Athens, Greece. Written informed consent was obtained from the patients. During that time, 24 [^18^F]FDG-PET/CT scans were performed on 22 patients. Among the scans, 15 were performed to assess for possible recurrence with equivocal findings in conventional imaging methods and 9 for restaging post-therapy, with 5 scans following surgery and 4 following chemotherapy. In all cases, conventional imaging studies were performed with at least one other imaging method 5–30 days prior to PET/CT, including 27 chest CT scans, 7 abdominal CT scans, 5 bone scans and 6 brain MRI scans.

### PET/CT procedure and technical details

A standard whole-body [^18^F]FDG-PET/CT approach was used for all patients. The patients were asked to fast for at least 6 h before the imaging study. The serum glucose concentration, measured prior to [^18^F]FDG administration, was <150 mg/dl in all patients (range, 61–145 mg/dl). Image acquisition started 1 h after the intravenous injection of a dose of 5 MBq/kg [^18^F]FDG. All acquisitions were performed using an integrated PET/CT scanner; the majority of scans used a Discovery ST (General Electric Medical Systems, Little Chalfont, UK), although three used a Biograph 6 (Siemens Medical Solutions, Erlangen, Germany). A whole-body image from the mid- femur to the base of the brain, typically divided into 6 bed positions, was obtained. The PET emission images were acquired with a 4-min acquisition period at each bed position. The imaging system enabled the simultaneous acquisition of 47 transverse PET images per field of view, using 3.27-mm intersection spacing, for an overall 15.7-cm transverse field of view. The PET resolution was ∼6.1 mm full width at half maximum near the centre of the field of view. A 4-detector row helical CT scanner (140 kV and 80 mA) was also included in the PET/CT system. The resulting CT images were used not only in image fusion, but also in the generation of an attenuation map for attenuation correction. A PET scan was acquired in the two-dimensional mode. The field of view and pixel size of the reconstructed images were 50 cm and 3.91 mm, respectively, with a matrix size of 128×128. The reconstruction method used was filtered back projection with a Hanning filter. PET/CT scans were interpreted visually by a nuclear medicine physician and a radiologist.

### Interpretation

Standard whole-body PET/CT images were reviewed on a Xeleris workstation in the transverse, coronal and sagittal planes, along with maximum intensity projection images. For visual analysis, [^18^F]FDG-PET/CT uptake was considered to be abnormal if located outside the normal anatomical structures or if the intensity was greater than the background blood-pool activity or adjacent normal tissue. In addition, the standardized uptake value (SUV) of the lesions was measured on the standard whole-body PET/CT in a semi-quantitative manner. SUV was calculated using the following formula:

$$SUV=\frac{Cdc}{\frac{di}{w}}.$$

where Cdc is the decay-corrected tracer tissue concentration (Bq/g), di is the injected dose (Bq) and w is the patient’s body weight (g). The maximum SUV (SUVmax) was recorded for each lesion after applying regions of interest (ROI) in the transaxial attenuation-corrected PET slices, around the pixels showing the greatest accumulation of [^18^F]FDG.

For diagnostic reasons, the lymph nodes were divided into the following groups: cervical, axillary, hilar and peribronchial, other mediastinal lymph nodes (precarinal, subcarinal, paratracheal, prevascular and aortopulmonary window) and abdominal. The SUVmax was defined from the lesion with the highest [^18^F]FDG uptake of each organ or every lymph node group.

### Data analysis

The imaging findings were classified as true-positive for local recurrence or metastasis if confirmed by one of the following criteria: a) positive histopathology results from biopsies or resections; or b) the presence of a detectable lesion at the corresponding site in follow-up conventional imaging studies.

### Statistical analysis

In order to compare the [^18^F]FDG uptake in the patients of the present study, [^18^F]FDG-PET/CT scans were randomly selected if they were performed between June and September 2011 for the detection of possible recurrence in patients with a history of NSCLC, with any type other than BAC. Statistical analysis was performed using the Statistical Package for Social Sciences (SPSS 17.0) software. Two-tailed paired t-tests were used to compare the categories of parameters. P<0.05 was considered to indicate statistically significant differences.

## Results

In total, 24 diagnostic [^18^F]FDG-PET/CT scans were performed in 22 patients and retrospectively reviewed. In all cases there was histological confirmation of BAC following surgical excision (lobectomy or sphenoid removal) in 21 patients and needle biopsy in the remaining patient. The study group consisted of 16 male and 6 female patients, with ages ranging between 49 and 78 years (mean, 65.1±8.9 years). PET/CT scans were repeated when there was a suspicion of new recurrence and the diagnosis was uncertain; thus, two patients underwent a second PET/CT scan, 10 and 13 months after the first, respectively. The study population characteristics are shown in Table I.

Among the 24 [^18^F]FDG-PET/CT scans, 18 were positive and 6 negative. Among the positive [^18^F]FDG-PET/CT scans, the lesions were located in lung nodules or masses (8 cases), local recurrence in the surgical margin (4 cases), pulmonary hilar and/or peribroncheal lymph nodes (6 cases), mediastinal lymph nodes, including precarinal, subcarinal, paratracheal, prevascular and aortopulmonary window (10 cases), cervical/supraclavicular lymph nodes (3 cases), axillary lymph nodes (2 cases), bones (2 cases), abdominal lymph nodes (1 case), adrenal gland (1 case), brain (1 case) and subcutaneous nodule (1 case).

Of the 18 positive scans, biopsies were performed in 9 cases and histological confirmation was achieved, while 5 of the remaining scans were classified as true-positive due to the presence of a detectable lesion at the corresponding site in follow-up conventional imaging studies. No data were available for the remaining 4 cases. In cooperation with the referral oncologists and after all the appropriate clinical and laboratory examinations, other possible pathologies for these patients were ruled out.

Among 15 patients examined with [^18^F]FDG-PET/CT to assess for suspected recurrence, the median follow-up between the initial diagnosis and surgery and the [^18^F]FDG-PET/CT scan was 33.45 months (range, 4 months to 8 years). Of the 15 scans performed for suspected recurrence, upstaging was observed in 9 and 2 were in agreement with the findings of the conventional modalities. A greater number of lesions was detected in 2 scans and fewer lesions were detected in one, with no change in staging, while one [^18^F]FDG-PET/CT scan was negative in a patient with equivocal findings in CT. The [^18^F]-FDG PET/CT scans detected 34 lesions and the mean SUVmax was 6.8±3.26 (range, 2.3–15.2).

**Figure 1:**
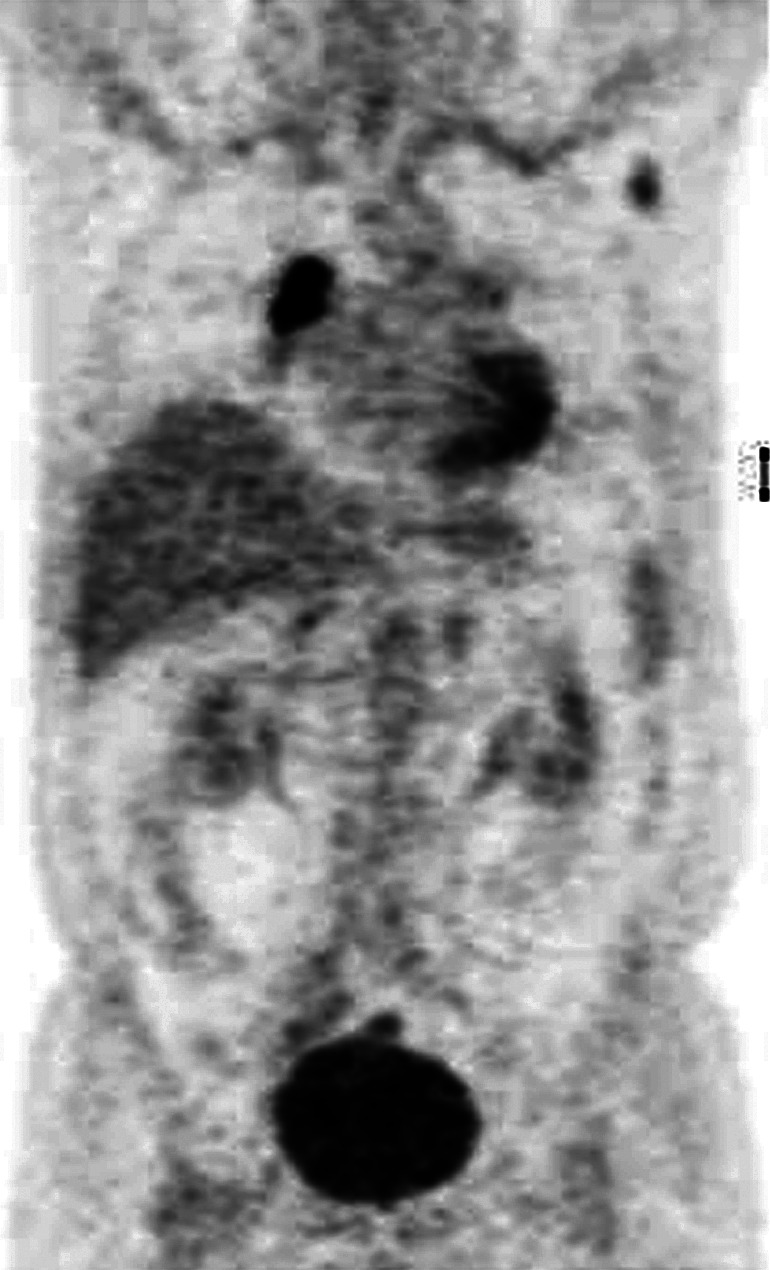
[^18^F]FDG-PET/CT image of a 74-year-old male patient, 4 months after the initial surgery. The [^18^F]FDG-PET/CT performed to assess for suspected recurrence showed increased uptake of [^18^F]FDG in the left axillary lymph nodes (SUVmax = 4.2), right hilar lymph nodes (SUVmax = 17.5) and lymph nodes in the anterior mediastinum, behind the sternum (SUVmax = 3.3). The previously performed CT had not correctly assessed the mediastinal lymph nodes due to their small size. FDG-PET/CT, fluorodeoxyglucose-positron emission tomography/computed tomography; SUVmax, maximum standardized uptake value.

**Figure 2:**
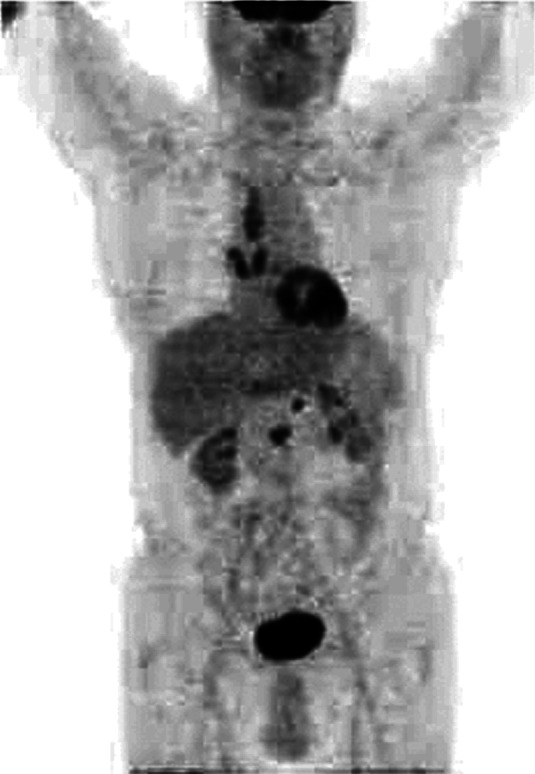
[^18^F]FDG-PET/CT image of a 67-year-old male patient, 2 years after the initial treatment. The [^18^F]FDG-PET/CT performed to assess for suspected recurrence showed increased uptake of [^18^F]FDG in the mediastinal lymph nodes (SUVmax = 6.1), including the right paratracheal, precarinal, subcarinal and right hilar lymph nodes (SUVmax = 7.9), a left superior mesenteric lymph node (SUVmax = 5.5) and the body of the second lumbar vertebra (SUVmax = 6.9). The previously performed CT had not revealed the lesions in the vertebra and the mesenteric lymph node. FDG-PET/CT, fluorodeoxyglucose-positron emission tomography/computed tomography; SUVmax, maximum standardized uptake value.

There were a number of cases where [^18^F]FDG-PET/CT imaging led to upstaging. The detection of mediastinal lymph nodes which were not detectable in previous CT scans led to upstaging from N0 or N1 to N2 (6 cases; Fig. 1, patient ID 3 Table I). The detection of distant metastases in the bone and abdominal lymph nodes resulted in upstaging from M0 to M1 (1 case; Fig. 2, patient ID 5 Table I), while in 2 cases [^18^F]FDG-PET/CT detected local recurrence and hilar lymph nodes, which were considered to be posttreatment rather than pathological lesions in CT. In patients where [^18^F]FDG-PET/CT detected a greater number of lesions with no change in staging, these findings were due to the detection of more mediastinal lymph nodes.

By contrast, among the patients examined for restaging following surgery or chemotherapy/radiotherapy, there was agreement between the findings of [^18^F]FDG-PET/CT and conventional modalities in 8 out of 9 cases. In 3 cases, the findings were in agreement with the findings of conventional modalities, while 5 patients had negative ^18^F-FDG PET/CT scans, which was in agreement with the negative conventional imaging performed the previous month. Consequently, there was discrepancy between the findings of [^18^F]FDG-PET/CT and the conventional imaging methods in only one patient. There were only 5 lesions with a mean SUVmax of 4.86±3.18 (range, 2.1–7.9).

## Discussion

Although the revised WHO definition strictly defines BAC as having absolutely no evidence of invasion, adenocarcinoma may display a range of BAC features due to predominant BAC with only a small focal area of invasion to a lesion that has BAC features only at the periphery of the tumor (12,13). Pure BAC, by definition, is not associated with lymph-node or distant metastases (13). The original feature of adenocarcinoma with BAC features is the intrapulmonary dissemination of the disease due to bronchial or lymphatic spread, leading to a high frequency of local or regional evolution and rare metastatic dissemination (13,14). Among patients with mixed BAC tumors, 10–25% have mediastinal nodal involvement and 5% present distant metastases (14).

All the patients included in the present study had known BAC, according to the referral oncologists, based on the histological examination following surgery. Taking into consideration the present data, we conclude that it is possible that certain patients had mixed BAC with adenocarcinoma components rather than pure BAC and that the pathologists who examined the tumors did not separate the mixed type from pure BAC. It is likely that the different classifications used, concerning the pathological discrimination between pure BAC and mixed type, are responsible for the large range in the rates of incidence of BAC among various series (4,15–17). Consequently, BAC is reported to account for 4–29% of NSCLC (4,15–17). Studies have suggested an increase in the incidence of BAC histology in the past 30 years (15,18). One explanation for this apparent increase is that pathologists have begun to report BAC features more frequently (4).

The present study showed that [^18^F]FDG-PET/CT was useful in the detection of suspected recurrence in patients with BAC and clinical symptoms and/or equivocal findings in conventional imaging methods, mainly CT. In 9 out of 15 cases (60%), the [^18^F]FDG-PET/CT scans upstaged the patients to a higher TNM stage, identifying sites of recurrence in organs or lymph nodes that were not detectable with previous conventional imaging methods and taking advantage of the whole-body scanning. By contrast, [^18^F]FDG-PET/CT did not appear to be as useful for examining patients to assess for restaging soon after surgery or chemotherapy. Only 1 case out of 9 (11%) had different results from the conventional imaging methods. This difference may be explained by the fact that if a tumor recurs, it is definitely more aggressive than a pure BAC.

Due to the usefulness of [^18^F]FDG-PET/CT in the detection of BAC recurrence, we decided to focus on this indication. Early detection of recurrence is important in order to make early decisions for the appropriate treatment. Several studies have been performed on the usefulness of [^18^F]FDG-PET and [^18^F]FDG-PET/CT in the detection of recurrence in the other subtypes of NSCLC, but not BAC, and have shown that this method is able to confirm or exclude tumor recurrence in cases with equivocal findings in CT, with a reported sensitivity of 97–100% and specificity of 62–100% (19–20). Similar results were observed in the present study for patients with BAC who underwent [^18^F]FDG-PET/CT to assess suspected recurrence.

In the literature, there are studies concerning the role of [^18^F]FDG-PET and [^18^F]FDG-PET/CT in the detection of BAC for the initial diagnosis, but not recurrence (21–25). The first of these studies was in 1998 and showed that [^18^F]FDG-PET was not able to initially detect BAC as the mean SUV in BAC was significantly lower compared with well- and poorly differentiated adenocarcinomas (22,23,25). Only approximately half of patients with BAC have a maximum SUV >2.5, the commonly accepted threshold for distinguishing benign from malignant lung lesions (14). This lower [^18^F]FDG uptake appears to be due to the low metabolic activity of BAC tumors and their slower proliferative activity in comparison with other types of lung cancer (21,22,25).

With regard to adenocarcinomas with a BAC component, a study that used the 1999 WHO definition demonstrated that the diagnostic accuracy of [^18^F]FDG-PET was similar between patients with adenocarcinoma with a BAC component and those with other types of NSCLC, with a sensitivity of ∼90% (21). However, metabolic imaging with [^18^F]FDG-PET was less accurate in patients with pure BAC lesions (100% BAC component) without invasive components (21,26). However, the mean SUVmax for adenocarcinoma with a BAC component was significantly lower compared with other types of NSCLC. The mean SUVmax for adenocarcinoma with a BAC component was 7.2 while it was 13.33 in other types of NSCLC (P<0.0001) (21). Additionally, a correlation was observed between [^18^F]FDG uptake and BAC components (P= 0.01) (21). The average SUV value of pure BAC was lower (SUVmax = 3.5) than in non-BAC adenocarcinoma (SUVmax = 8.8) and squamous cell carcinoma (SUVmax = 10.8) (22).

These findings are similar to the results of the present study. The mean SUVmax of the 34 sites of recurrence with [^18^F]FDG uptake was 6.8±3,26. In order to compare this uptake, the [^18^F]FDG-PET/CT scans of 29 patients, performed between June and September 2011 to assess suspected recurrence of NSCLC of any type except BAC, were randomly selected. In total, 17 patients with adenocarcinoma and 12 with squamous cell lung cancer were studied. The mean SUVmax of the 49 sites of recurrence in this group of patients was 9.1±5.5. It is clear that the [^18^F]FDG uptake of BAC recurrence sites was lower than those of the other types of NSCLC. An independent-sample t-test was performed to compare the SUVmax of the two groups of patients. This showed that there was a statistically significant difference in the scores for BAC (mean, 6.8; SD, 3.26) and other types of NSCLC (mean, 9.1; SD, 5.50); t(79)=−2.3, P=0.022.

The lower [^18^F]FDG uptake of BAC and adenocarcinomas with a BAC component according to certain studies may occur due to different levels and extents of expression of the glucose transporter, Glut-1 (27). It is known that facilitative glucose transport across the plasma membrane is mediated by a family of structurally related proteins, known as facilitated diffusion glucose transporters, or Gluts (28). Glut-1 is the major glucose transporter expressed in NSCLC (28). Glut-1 expression correlates with [^18^F]FDG uptake and has an essential role in high [^18^F]FDG accumulation. The degree of cell differentiation is correlated with Glut-1 expression and [^18^F]FDG uptake in adenocarcinoma of the lung, with increased expression of Glut-1 associated with a lesser degree of differentiation (28). The rate of glucose uptake via glucose transporter is regulated under conditions associated with cell proliferation, differentiation or transformation (28). Changes in the rates of glucose uptake and the overexpression of glucose transporters are also associated with adaptation to hypoxia, partly due to increased dependency on glycolysis as an energy source, a condition that may arise in rapidly growing tumors (29). Consequently, Glut expression reflects the biological behavior of cancer cells. It is reported that enhanced [^18^F]FDG uptake via the overexpression of Glut-1 may be associated with aggressive tumor behavior and poor prognosis for NSCLC. Glut-1 expression and [^18^F]FDG uptake were significantly lower in BAC than in non-BAC cases (28). A previous study showed that 85.7% of BACs were negative for Glut-1 expression in histological examinations, while the corresponding value for non-BAC adenocarcinomas was only 4.3% (P<0.0001) (28).

As has already been mentioned, BAC is a source of false-negative findings in [^18^F]FDG-PET/CT imaging. Several studies have been performed using radiopharmaceuticals that follow the lipid metabolism, such as ^11^C or ^18^F-choline (30–32). Choline is an essential component of the phospholipids in the cell membrane. Cancer cells are able to actively incorporate choline to produce phosphatidyl choline to facilitate cancer cell duplication. Certain malignant tumors show a high proliferation rate with an increased cell membrane metabolism that leads to increased choline uptake (30). However, until now, studies have revealed no superiority of ^11^C or ^18^F-choline over [^18^F]FDG-PET/CT for detecting lesions with a BAC component.

In conclusion, the present study indicates that in patients with known lung cancer diagnosed as BAC, imaging with [^18^F]FDG-PET/CT was able to offer more information concerning the detection of recurrence sites, with high accuracy. The [^18^F]FDG uptake of BAC, as measured with the semi-quantitative factor SUVmax, is lower than the uptake of the other types of NSCLC but high enough to contribute to the accurate detection of recurrence. Although it is known from several studies that [^18^F]FDG-PET/CT has a low sensitivity with regard to the initial diagnosis of BAC, there are no other published data on the role of [^18^F]FDG-PET/CT in the detection of recurrence for this type of cancer.

## Figures and Tables

**Table I:** Patient characteristics and [^18^F]FDG-PET/CT findings.

Patient ID	Age (years)/gender	Reason for reference	[^18^F]FDG-PET/CT findings	SUVmax	Comparison [^18^F] FDG-PET/CT with CT
1	55/F	Recurrence	Mediastinal lymph nodes	2.4	Fewer lesions with no change in staging
			Brain	7.0	
2	65/M	Recurrence	Negative	-	Downstaging
3	74/M	Recurrence	Lymph nodes:		Upstaging
			Lung hilar	17.5	
			Mediastinal	3.3	
			Axillary	4.2	
4a	58/F	Restaging after chemotherapy	Cervical lymph nodes	3.0	Same findings
4b		Restaging after chemotherapy	Negative	-	Same findings
5	67/M	Recurrence	Lymph nodes:		Upstaging
			Hilar/peribroncheal	6.1	
			Mediastinal	7.9	
			Abdominal	5.5	
			Bones	6.9	
6	78/F	Recurrence	Pulmonary nodule	3.7	Same findings
7	58/M	Restaging after chemotherapy	Negative	-	Same findings
8	67/M	Recurrence	Multiple pulmonary nodules	4.7	More lesions with no change in staging
			Lung hilar lymph nodes	7.0	
			Mediastinal lymph nodes	7.5	
9a	69/M	Restaging after chemotherapy	Negative	-	Same findings
9b		Recurrence	Local recurrence	5.7	Fewer lesions with no change in staging
			Mediastinal lymph nodes	6.7	
			Bones	9.7	
			Adrenal gland	5.0	
10	59/F	Restaging after surgery	Negative	-	Same findings
11	49/M	Restaging after surgery	Pulmonary nodule	3.4	Upstaging
			Mediastinal lymph nodes	5.8	
12	70/F	Recurrence	Pulmonary nodule	10.0	Upstaging
			Mediastinal lymph nodes	6.0	
13	73/M	Recurrence	Local recurrence	9.8	Upstaging
14	52/M	Restaging after chemotherapy	Negative	-	Same findings
15	76/M	Recurrence	Cervical/supraclavicular lymph nodes	3.8	Upstaging
			Axillary lymph nodes	11.5	
			Subcutaneous nodule	2.6	
16	69/M	Recurrence	Local recurrence	7.5	Same findings
			Pulmonary nodules	9.0	
17	75/M	Recurrence	Lymph nodes:		
			Peribroncheal	4.7	Upstaging
			Mediastinal	4.0	
18	55/M	Recurrence	Lung hilar lymph nodes	3.8	Upstaging
19	77/M	Restaging after chemotherapy	Pulmonary nodule	2.1	Same findings
20	60/M	Restaging after chemotherapy	Pulmonary mass	10.0	Same findings
21	54/F	Recurrence	Pulmonary nodules	2.3	Upstaging
			Local recurrence	10.5	
			Mediastinal lymph nodes	5.5	
22	72/M	Recurrence	Lymph nodes:		
			Cervical/supraclavicular,	10.8	Upstaging
			Lung hilar	11.3	
			Mediastinal	7.4	

FDG-PET/CT, fluorodeoxyglucose-positron emission tomography/computed tomography; SUVmax, maximum standardized uptake value; M, male; F, female.

## References

[1] Alberg AJ, Ford JG, Samet JM, American College of Chest Physicians (2007). Epidemiology of lung cancer: ACCP evidence-based clinical practice guidelines (2nd edition). Chest.

[2] Barkley JE, Green MR (1996). Bronchioloalveolar carcinoma. J Clin Oncol.

[3] Yousem SA, Beasley MB (2007). Bronchioloalveolar carcinoma: a review of current concepts and evolving issues. Arch Pathol Lab Med.

[4] Raz DJ, He B, Rosell R, Jablons DM (2006). Bronchioloalveolar carcinoma: a review. Clin Lung Cancer.

[5] Lee HY, Lee KS, Han J, Kim BT, Cho YS, Shim YM, Kim J (2009). Mucinous versus nonmucinous solitary pulmonary nodular bronchioloalveolar carcinoma: CT and FDG PET findings and pathologic comparisons. Lung Cancer.

[6] Beasley MB, Brambilla E, Travis WD (2005). The 2004 World Health Organization classification of lung tumors. Semin Roentgenol.

[7] Pauwels EK, Sturm EJ, Bombardieri E, Cleton FJ, Stokkel MP (2000). Positron-emission tomography with [^18^F]fluorodeoxyglucose. Part I Biochemical uptake mechanism and its implication for clinical studies. J Cancer Res Clin Oncol.

[8] Alavi A, Reivich M (2002). Guest editorial: the conception of FDG-PET imaging. Semin Nucl Med.

[9] Arenberg D (2011). Bronchioloalveolar carcinoma. Semin Respir Crit Care Med.

[10] Takashima S, Maruyama Y, Hasegawa M, Saito A, Haniuda M, Kadoya M (2003). High-resolution CT features: prognostic significance in peripheral lung adenocarcinoma with bronchioloalveolar carcinoma components. Respiration.

[11] Sung YM, Lee KS, Kim BT, Han J, Lee EJ (2005). Lobar mucinous bronchioloalveolar carcinoma of the lung showing negative FDG uptake on integrated PET/CT. Eur Radiol.

[12] Zell JA, Ou SH, Ziogas A, Anton-Culver H (2005). Epidemiology of bronchioloalveolar carcinoma: improvement in survival after release of the 1999 WHO classification of lung tumors. J Clin Oncol.

[13] Liu S, Cheng H, Yao S, Wang C, Han G, Li X, Liu C (2010). The clinical application value of PET/CT in adenocarcinoma with bronchioloalveolar carcinoma features. Ann Nucl Med.

[14] Raz DJ, Kim JY, Jablons DM (2007). Diagnosis and treatment of bronchioloalveoral carcinoma. Curr Opin Pulm Med.

[15] Barsky SH, Cameron R, Osann KE, Tomita D, Holmes EC (1994). Rising incidence of bronchioloalveolar lung carcinoma and its unique clinicopathologic features. Cancer.

[16] Donker R, Stewart DJ, Dahrouge S, Evans WK, Shamji FM, Maziak DE, Tomiak EM (2000). Clinical characteristics and the impact of surgery and chemotherapy on survival of patients with advanced and metastatic bronchioloalveolar carcinoma: a retrospective study. Clin Lung Cancer.

[17] Read WL, Page NC, Tierney RM, Piccirillo JF, Govindan R (2004). The epidemiology of bronchioloalveolar carcinoma over the past two decades: analysis of the SEER database. Lung Cancer.

[18] Travis WD, Garg K, Franklin WA (2005). Evolving concepts in the pathology and computed tomography imaging of lung adenocarcinoma and bronchioloalveolar carcinoma. J Clin Oncol.

[19] Schrevens L, Lorent N, Dooms C, Vansteenkiste J (2004). The role of PET scan in diagnosis, staging, and management of non-small cell lung cancer. Oncologist.

[20] Hicks RJ, Kalff V, MacManus MP, Ware RE, McKenzie AF, Matthews JP, Ball DL (2001). The utility of ^18^F-FDG PET for suspected recurrent non-small cell lung cancer after potentially curative therapy: impact on management and prognostic stratification. J Nucl Med.

[21] Yap CS, Schiepers C, Fishbein MC, Phelps ME, Czernin J (2002). FDG-PET imaging in lung cancer: how sensitive is it for bronchioloalveolar carcinoma?. Eur J Nucl Med Mol Imaging.

[22] Kim BT, Kim Y, Lee KS (1998). Localized form of bronchioloalveolar carcinoma: FDG PET findings. AJR Am J Roentgenol.

[23] Smith GT, Hubner KF, Peterson A, Hunter K, Neff J (1998). FDG PET for evaluation of bronchioloalveolar cell carcinoma (BAC) of the lung. Clin Positron Imaging.

[24] Heyneman LE, Patz EF (2002). PET imaging in patients with bronchioloalveolar cell carcinoma. Lung Cancer.

[25] Higashi K, Ueda Y, Seki H (1998). Fluorine-18-FDG PET imaging is negative in bronchioloalveolar lung carcinoma. J Nucl Med.

[26] Sun JS, Park KJ, Sheen SS, Yoon JK, Yoon SN, Lee KB, Hwang SC (2009). Clinical usefulness of the fluorodeoxyglucose (FDG)-PET maximal standardized uptake value (SUV) in combination with CT features for the differentiation of adenocarcinoma with a bronchioloalveolar carcinoma from other subtypes of non-small cell lung cancers. Lung Cancer.

[27] Khandani AH, Whitney KD, Keller SM, Isasi CR, Donald Blaufox M (2007). Sensitivity of FDG PET, GLUT1 expression and proliferative index in bronchioloalveolar lung cancer. Nucl Med Commun.

[28] Higashi K, Ueda Y, Sakurai A (2000). Correlation of Glut-1 glucose transporter expression with [^18^F]FDG uptake in non-small cell lung cancer. Eur J Nucl Med.

[29] Vaupel P, Kallinowski F, Okunieff P (1989). Blood flow, oxygen and nutrient supply, and metallic microenvironment of human tumors: a review. Cancer Res.

[30] Balogova S, Huchet V, Kerrou K (2010). Detection of bronchioalveolar cancer by means of PET/CT and ^18^F-fluorocholine, and comparison with ^18^F-fluorodeoxyglucose. Nucl Med Commun.

[31] Higashi K, Ueda Y, Matsunari I (2004). ^11^C-acetate PET imaging of lung cancer: comparison with ^18^F-FDG PET and ^99m^Tc-MIBI SPET. Eur J Nucl Med Mol Imaging.

[32] Shibata H, Nomori H, Uno K (2009). ^11^C-acetate for positron emission tomography imaging of clinical stage IA lung adenocarcinoma: comparison with ^18^F-fluorodeoxyglucose for imaging and evaluation of tumor aggressiveness. Ann Nucl Med.

